# Comparison of mNGS and conventional culture in non-organ transplant critically ill patients supported by ECMO: a single-center study

**DOI:** 10.3389/fcimb.2023.1146088

**Published:** 2023-04-17

**Authors:** Xi Zhao, Lin-Peng Bai, Bo-Yan Li, Zhen-Zhen Yue, Yang-Chao Zhao, Xiao-Yan Zhao

**Affiliations:** ^1^ Department of Cardiology, Cardiovascular Center, Henan Key Laboratory of Hereditary Cardiovascular Diseases, The First Affiliated Hospital of Zhengzhou University, Zhengzhou, Henan, China; ^2^ Department of Extracorporeal Life Support Center, Department of Cardiac Surgery, The First Affiliated Hospital of Zhengzhou University, Zhengzhou, Henan, China

**Keywords:** metagenomic next-generation sequencing, conventional culture, extracorporeal membrane oxygenation, non-organ transplant, different therapy periods

## Abstract

**Objectives:**

Infection is one of the important causes of death in intensive care unit (ICU) patients. At present, there are few articles focused on the detailed analysis of pathogenic microorganisms detected in different therapy periods of critically ill patients supported by extracorporeal membrane oxygenation (ECMO).

**Methods:**

From October 2020 to October 2022, ECMO-assisted patients who underwent multiple times of both metagenomic next-generation sequencing (mNGS) test and conventional culture were enrolled continuously in the First Affiliated Hospital of Zhengzhou University. The baseline data, laboratory test results, and pathogenic microorganisms detected by mNGS and traditional culture in different time periods were recorded and analyzed.

**Results:**

In the present study, 62 patients were included finally. According to whether the patients survived at discharge, they were divided into the survivor group (n = 24) and the non-survivor group (n = 38). Then, according to the different types of ECMO support, they were divided into the veno-venous ECMO (VV ECMO) group (n = 43) and the veno-arterial ECMO (VA ECMO) group (n = 19). The summit period of specimens of traditional culture and mNGS detection of ECMO patients was 7 days after admission, and the largest number of specimens of surviving patients appeared after ECMO withdrawal. The total number of traditional culture specimens was 1,249, the positive rate was 30.4% (380/1,249), and the positive rate of mNGS was 79.6% (82/103). A total of 28 kinds of pathogenic microorganisms were cultured from conventional culture, and 58 kinds of pathogenic microorganisms were detected by mNGS, including *Mycobacterium*, *Rickettsia*, and *Chlamydia psittaci*. In conventional culture, the most frequent Gram-negative bacteria, Gram-positive bacteria, and fungi were *Klebsiella pneumoniae*, *Corynebacterium striatum*, and *Candida glabrata*, and those with the highest frequency of occurrence in mNGS detection were *Acinetobacter baumannii*, *Enterococcus faecium*, and *Aspergillus flavus*.

**Conclusions:**

Throughout the whole treatment process, different kinds of suspicious biological specimens of high-infection-risk ICU patients supported by ECMO should undergo both mNGS detection and traditional culture early and repeatedly.

## Introduction

Extracorporeal membrane oxygenation (ECMO), also known as extracorporeal life support, is an important mechanism for the treatment of critical illness. It is typically divided into veno-venous ECMO (VV ECMO) and veno-arterial ECMO (VA ECMO) based on the types of blood vessels drained and reinfused and the types of organs supported (Professional Committee for Extracorporeal Life Support of Chinese Medical Doctor Association, 2018). ECMO acts as a bridge between critical illnesses (e.g., cardiogenic shock, septic shock, and respiratory failure) and further treatment or decision. Moreover, because it can quickly and effectively replace vital organ functions, ECMO is considered a last resort to save lives in emergency rescue (Ventetuolo and Muratore, 2014; Guglin et al., 2019). Nowadays, ECMO is widely used in the intensive care unit (ICU). Timely and correct application of ECMO can effectively improve the survival rate of severe patients and even improve the prognosis (Oh et al., 2021; Kochanek et al., 2022). However, the infection rate of severe patients should not be underestimated. An invasive procedure is one of the important risk factors of infection in severe patients, including continuous renal replacement therapy (CRRT), intra-aortic balloon pump counterpulsation (IABP), deep venipuncture, tracheal intubation, and even ECMO cannulas (Kühn et al., 2020). Infections are common complications in ECMO patients and are associated with an increase in mortality (Burket et al., 1999). Early and accurate identification of pathogenic microorganisms is the key to the treatment of infection.

Traditional bacterial culture has been used in clinical practice for many years, which can cultivate pathogenic bacteria and conduct drug sensitivity tests. However, ECMO patients often suffer from mixed infections and special pathogen infections due to long hospital stays and physical weakness. At this time, a traditional bacterial culture is limited. Metagenomic next-generation sequencing (mNGS) technology is widely used to explore the pathogens implicated in various infectious diseases by testing the DNA of all microorganisms directly extracted from clinical specimens (Zhang et al., 2020; Yue et al., 2021). The advantages of mNGS detection are high efficiency and accuracy and being a non-culture-based method. Recently, mNGS has been applied to pathogen detection in infectious diseases as a universal method to sequence and identify nucleic acids from microorganisms. In addition to high efficiency and accuracy, the advantages of mNGS include being a non-culture-based method.

At present, few literature comprehensively and detailedly analyzes the detection of pathogens using both mNGS and traditional culture in ECMO patients at different hospitalization and treatment periods. In the present study, we analyzed the data from 62 non-organ transplant critically ill patients supported by ECMO in the First Affiliated Hospital of Zhengzhou University.

## Materials and methods

### Patients and study design

The present study retrospectively enrolled ECMO-assisted patients who were detected to have pathogenic microorganisms by both mNGS and traditional culture from October 2020 to October 2022 at the First Affiliated Hospital of Zhengzhou University, respectively. The inclusion criteria were as follows: 1) the necessity to use ECMO, 2) patients had completed ECMO treatment (more than 48 h), and 3) detected pathogenic microorganisms by both mNGS and traditional culture at least once. The exclusion criteria were as follows: 1) aged <18 years, 2) pregnant, and 3) ECMO used to assist organ transplantation operation. During this period, 152 patients who had indications for ECMO assistance were detected to have pathogenic microorganisms by both mNGS and traditional culture. Among them, 84 organ transplantation patients and six patients with ECMO assistant time less than 48 h were excluded. Finally, 62 patients, including 46 men and 16 women, were enrolled and divided into the non-survivor group (n = 38) and the survivor group (n = 24) according to their discharged status. Then, based on the type of ECMO assistance, patients were divided into the VV ECMO group (n = 43) and the VA ECMO group (n = 19).

The baseline characteristics, clinical data, and pathogens of 62 critically ill patients assisted by ECMO were recorded and analyzed. The clinical data of the ECMO patients were obtained through the electronic medical record system. We used Sequential Organ Failure Assessment (SOFA), Acute Physiology and Chronic Health Evaluation II (APACHE II) scores, and the D-values of SOFA and APACHE II scores to evaluate the patients’ condition. The D-values of SOFA and APACHE II scores were defined as the highest score of 48 h of ECMO assistance subtracted from the highest score before ECMO assistance.

The present study was approved by the Ethics Committee of the First Affiliated Hospital of Zhengzhou University, Zhengzhou, China (no. 2020-KY-429). All procedures performed in this study involving human participants were in accordance with the Declaration of Helsinki. All participants provided written informed consent before participation.

### ECMO application

ECMO equipment adopts an ECMO system kit, including a centrifugal pump, oxygenator, and its connecting pipeline, among which the centrifugal pump adopts Rotaflow pump head and controller from Medtronic and MAQUET. The oxygenator is divided into Medtronic and MAQUET adult oxygenators. The pipeline includes an ECMO kit pipeline and an ordinary PVC pipeline, and the cannulas are all arteriovenous thin-walled cannulas. The commonly used site for patients assisted by VV ECMO is the femoral vein–internal jugular vein, and the puncture method was percutaneous venous catheterization. In VA ECMO patients, sites of vascular access were the femoral vessels.

VV ECMO management 1) Management of mechanical ventilation: to avoid or reduce the occurrence of ventilator-related lung injury to the greatest extent while promoting the recruitment of collapsed alveoli. 2) Anticoagulation: unfractionated heparin anticoagulation to maintain activated coagulation time (ACT) is 1.5 times the upper limit of normal, and the activated partial thromboplastin time (APTT) is 40–55 s. 3) Analgesia and sedation: adequate analgesia, sedation combined with muscle relaxants, combined with the patients’ human–machine synchronization, and the complete suppression of spontaneous breathing. 4) Flow management: adjust the flow according to the patients’ monitoring indicators to maintain blood oxygen saturation (SaO_2_) at 85%–95% and PaO_2_ above 60 mmHg; VV ECMO pump flow to 2–3 L/min, close VV ECMO airflow, under certain respiratory support (FiO_2_ < 50%, positive end-expiratory pressure (PEEP) ventilation ≤10 cmH_2_O, and peak airway pressure <30 cmH_2_O), monitor for 2–4 h, and if SaO_2_ > 95% and PaCO_2_ < 50 mmHg, weaning of VV ECMO can be considered.

In VA ECMO-assisted patients, the prompt number of revolutions per minute of the ECMO device was adjusted to meet the standard that the patients’ cardiac index of greater than 2.2 L/min/m^2^, central mixed venous oxygen saturation above 70%, and the mean arterial pressure above 65 mmHg. Usually, an arterial catheter was used to continuously monitor blood pressure. Weaning of VA ECMO can be considered if a flow of 0.5–1.0 L/min is achieved. Every stage of reductions in blood flow must be accessed by echocardiographic and hemodynamic assessments.

### Methods and process of mNGS

The samples were collected from the ECMO patients based on clinically therapeutic requirements. About 5 ml of sample was used to extract DNA by TIANamp Micro DNA Kit following the manufacturer’s instructions. DNA libraries were constructed by DNA fragmentation, end-repair, adapter ligation, and PCR amplification, followed by sequencing. Agilent 2100 Bioanalyzer (Agilent, USA) and ABI StepOnePlus Realtime PCR System were used for quality control of the DNA library; the qualified libraries were sequenced on NextSeq 550Dx platform (Illumina, USA) using 75-bp sequencing read length.

Illumina NextSeq 550 sequencer was used for the metagenomics sequence, and 15–20 samples that contain one negative control were loaded in each metagenomics sequencing batch. The internal reference, which is from *Arabidopsis thaliana*, was provided by sequencing manufacturers. High-quality sequencing data were generated by removing low-quality and short reads (<35 bp in length) and then yielding reads strictly aligned to pathogen species (SDSMRN) and reads strictly aligned to pathogen genus (SDSMRNG). The list of microorganisms obtained through the above analysis process was compared with an internal background database containing microorganisms present in more than 50% of samples in the laboratory over the past 3 months. Suspected background microorganisms were removed. Microorganisms with SDSMRN > 50 and at least three times higher than the control group were considered suspected pathogens, while the SDSMRN of suspected pathogens with SDSMRN < 50 should be at least five times higher than that of the control group.

The data that support the findings of this study have been deposited into the EMBL database with accession number PRJEB57970.

### Statistical analysis

All collected data were statistically analyzed using SPSS 21.0 (IBM Corp., Armonk, NY, USA). Variables of the two groups were compared using the Wilcoxon rank-sum test for continuous variables and the chi-square test for binary and categorical variables, as appropriate. p < 0.05 indicates that the difference is statistically significant.

## Results

### Characteristics of ECMO patients

**Figure 1:**
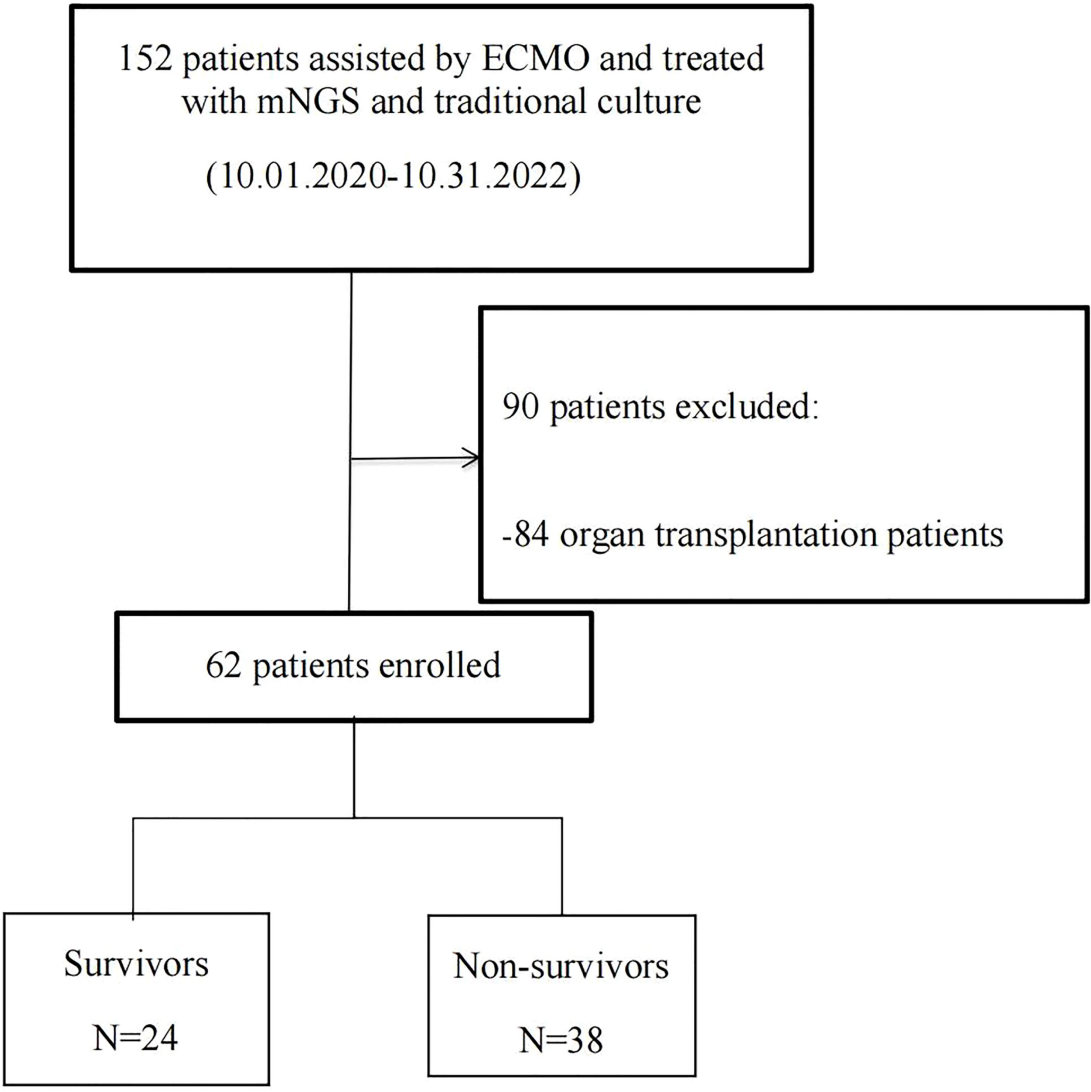
Flowchart of analyzed patients. ECMO, extracorporeal membrane oxygenation; mNGS, metagenomic next-generation sequencing.

The flowchart of this experiment is shown in **Figure 1**. As shown in **Table 1**, the survival ECMO patients were younger than the non-survivors (60 ± 14 *vs.* 49 ± 17 years, p = 0.008, **Table 1**). In general, the primary diseases of ECMO patients who underwent detection by mNGS and traditional culture were infectious shock (46.8%), respiratory failure (35.5%), and cardiogenic shock (11.3%). Before ECMO assistance, there was no significant difference between the survivors and non-survivors in the improvement of the condition evaluated by the two scoring systems (SOFA and APACHE II). Similarly, there was no significant difference between the two groups after 48 h of ECMO assistance in the improvement of the condition evaluated by the SOFA scores. However, the non-survivor group scored higher than the survivor group as evaluated by the APACHE II scores after 48 h of ECMO assistance (23.42 ± 7.277 *vs.* 18.58 ± 6.520, p = 0.010) (**Table 1**). Within 48 h of ECMO support, there was no significant difference between the two groups in the improvement of the condition as evaluated by the two scoring systems (SOFA and APACHE II) (**Table 1**). Among the enrolled patients, the support type of VA ECMO (30.6%) was significantly less than that of VV ECMO (69.4%), but there was no significant difference between the survivors and non-survivors. In addition, in terms of invasive operations related to infection, there was no statistical difference between the two groups in CRRT use rate, IABP use rate, ECMO support time, and mechanical ventilation time (**Table 1**).

**Table 1:** Baseline characteristics of ECMO patients.

Variables	Non-survivors (n = 38)	Survivors (n = 24)	p-Value
**Male** (n%)	31 (81.6)	15 (62.5)	0.118
Age* (years)	60 ± 14	49 ± 17	0.008
**BMI** (kg/m^2^)	23.8 ± 2.6	25.5 ± 9.2	0.304
**Comorbidities** (n%)			
Hypertension	7 (18.4)	10 (41.7)	0.078
Type 2 diabetes	8 (21.1)	4 (16.7)	0.670
Coronary heart disease	6 (15.8)	3 (12.5)	0.720
**Primary disease** (n%)			0.088
Septic shock	20 (52.6)	9 (37.5)	
Respiratory failure	15 (39.5)	7 (29.2)	
Cardiogenic shock	2 (5.3)	5 (20.8)	
Others	1 (2.6)	3 (12.5)	
**CRRT** (n%)	17 (44.7)	12 (50)	0.692
**IABP** (n%)	1 (2.6)	3 (12.5)	0.191
**Baseline SOFA and APACHE II scores**			
**SOFA scores (before** ECMO assistance**)**	9.23 ± 4.224	9.82 ± 4.231	0.648
**SOFA scores (48 h** of ECMO assistance**)**	11.97 ± 4.290	11.67 ± 4.622	0.791
**APACHE II scores (before** ECMO assistance**)**	21.43 ± 8.190	22.18 ± 9.098	0.775
**APACHE II scores (48 h** of ECMO assistance**)**	23.42 ± 7.277	18.58 ± 6.520	0.010
**SOFA D-value**	2 (0–4)	0 (–2–3)	0.102
**APACHE II D-value**	0 (–5–7)	0 (–12–1)	0.147
**ECMO type**			0.721
VV ECMO	27 (71.1)	16 (66.7)	
VA ECMO	11 (28.9)	8 (33.3)	
ECMO assistance **time** (h)	203 (123–304)	133 (87–189)	0.053
**Mechanical ventilation time** (h)	293 (219–444)	220 (145–443)	0.368

The data are shown as the mean ± SD, median (interquartile 25-75), or n (percentage).

ECMO, extracorporeal membrane oxygenation; BMI, body mass index; CRRT, continuous renal replacement therapy; IABP, intra-aortic balloon pump; SOFA, Sequential Organ Failure Assessment; APACHE, Acute Physiology and Chronic Health Evaluation.

^*^ Significant difference (p < 0.05).

### Laboratory examinations of ECMO patients

There was no significant difference in the liver (alanine transaminase (ALT) and aspartate transaminase (AST)) and kidney function (creatinine (Cr)), coagulation function (prothrombin time (PT) and APTT), and heart function (N-terminal pro-B-type natriuretic peptide (NT-pro-BNP)) between the non-survivor group and the survivor group within 48 h after ECMO support or on admission (**Tables 2**, **3**). Compared with baseline levels of lactate, the data showed an increase in lactate levels within 48 h after ECMO assistance. In terms of inflammation indicators, we selected blood leukocyte, procalcitonin, C-reactive protein, interleukin (Mandawat and Rao, 2017; Kühn et al., 2020), interferon-γ, and CD4^+^ T-cell absolute count for comparison, but no statistical difference was observed between the two groups during the two time periods (**Tables 2**, **3**).

**Table 2:** Baseline laboratory examinations of ECMO patients.

Laboratory examinations	Non-survivors (n = 38)	Survivors (n = 24)	p-Value
**WBC** (10^9^/L)	14.8 ± 7.4	12.5 ± 7.4	0.310
**Hb** (g/L)	119.1 ± 24.4	117.1 ± 24.6	0.798
**PLT** (10^9^/L)	160.3 ± 91.1	168 ± 75.6	0.770
**ALT** (U/L)	32 (21–61)	49 (11–176)	0.312
**AST** (U/L)	38 (27–118)	95 (64–125)	0.311
**Cr** (μmol/L)	66 (57–121)	95 (64–135)	0.740
**PT** (s)	13.7 ± 2	13.6 ± 3.8	0.875
**APTT** (s)	33.4 ± 15.2	41.4 ± 33.7	0.275
**NT-pro-BNP** (pg/ml)	2,170 (506–4,960)	178 (78–3,227)	0.627
**Lactate** (mmol/L)	2 (1.4–4.7)	2.5 (1.5–5.6)	0.812
**PCT** (ng/ml)	0.4 (0.2–3)	1.5 (0.2–29.6)	0.273
**CRP** (mg/L)	123.3 (53.5–205.3)	54.3 (11.61–171.6)	0.871
**IL-6** (pg/ml)	103.7 (45.9–217)	64.9 (10.8–80.5)	0.075
**IL-10** (pg/ml)	8 (6–16.5)	12.8 (5.2–27.8)	0.577
**IFN-γ** (pg/ml)	2.1 (1–3.2)	2 (1–9.1)	0.622
**CD4^+^ T-cell absolute count** (/μl)	142.7 (59.5–241.7)	256.8 (70.9–556.1)	0.866

The data are shown as the mean ± SD or median (interquartile 25-75).

ECMO, extracorporeal membrane oxygenation; WBC, white blood cell; Hb, hemoglobin; PLT, platelet; ALT, alanine transaminase; AST, aspartate transaminase; Cr, creatinine; PT, prothrombin time; APTT, activated partial thromboplastin time; PCT, procalcitonin; CRP, C-reactive protein; IL, interleukin; IFN, interferon.

**Table 3:** Laboratory examinations of ECMO patients within 48 h after ECMO assistance.

Laboratory examinations	Non-survivors (n = 38)	Survivors (n = 24)	p-Value
**WBC** (10^9^/L)	12.4 ± 7.1	11.7 ± 6.8	0.679
**Hb** (g/L)	93.6 ± 17.1	94.8 ± 21	0.809
**PLT** (10^9^/L)	104.7 ± 75.2	121.4 ± 87.4	0.427
**ALT** (U/L)	29 (18–130)	54 (31–169)	0.371
**AST** (U/L)	51 (29–156)	94 (39–308)	0.420
**Cr** (μmol/L)	70 (57–132)	105 (76–174)	0.356
**PT** (s)	16.1 ± 3.5	16.6 ± 4.4	0.577
**APTT** (s)	49.5 ± 31.4	55.2 ± 35	0.512
**NT-pro-BNP** (pg/ml)	1,710 (587–6,279)	2,350 (277–4,871)	0.948
**Lactate** (mmol/L)	2.9 (2.1–4.5)	4.6 (2–8.2)	0.131
**PCT** (ng/ml)	3.8 (1.2–14.1)	3.3 (0.8–31.1)	0.629
**CRP** (mg/L)	150 (70.4–213.7)	93.5 (39.5–191.2)	0.087
**IL-6** (pg/ml)	77.1 (7.8–464.2)	42.5 (13–443.8)	0.810
**IL-10** (pg/ml)	4.3 (2.8–18.8)	18.4 (7.7–63.5)	0.893
**IFN-γ** (pg/ml)	1.4 (0.6–3.9)	1.5 (1.2–2.2)	0.464
**CD4^+^T cell absolute count** (/μl)	s233.7 (84.6–426.1)	135.4 (102.1–454.2)	0.615

The data are shown as the mean ± SD or median (interquartile 25-75).

ECMO, extracorporeal membrane oxygenation; WBC, white blood cell; Hb, hemoglobin; PLT, platelet; ALT, alanine transaminase; AST, aspartate transaminase; Cr, creatinine; PT, prothrombin time; APTT, activated partial thromboplastin time; PCT, procalcitonin; CRP, C-reactive protein; IL, interleukin; IFN, interferon.

### Samples of mNGS and traditional culture

**Figure 2:**
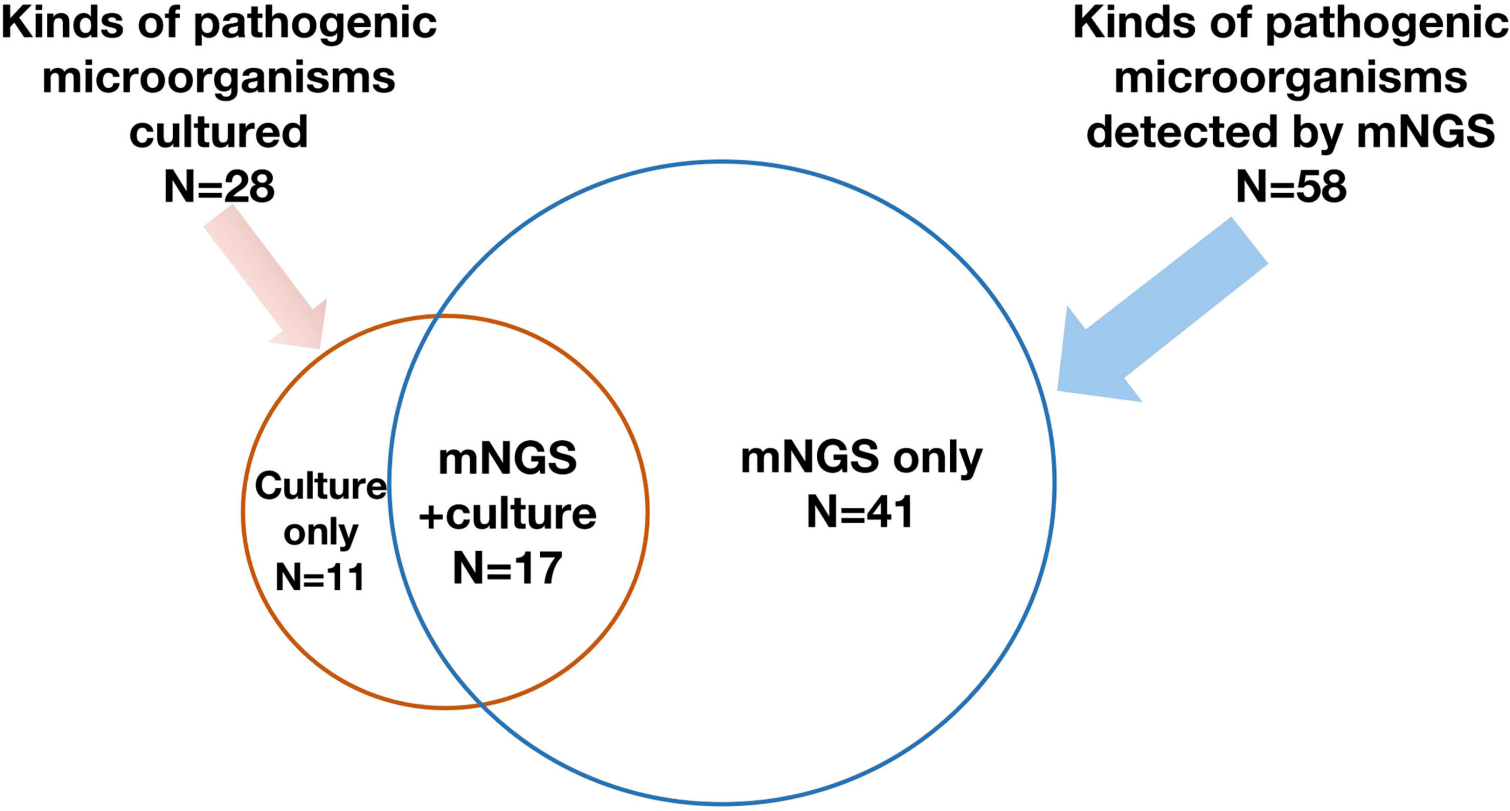
A total of 58 kinds of pathogenic microorganisms were detected by mNGS, while 28 of them were cultured from conventional culture, and 17 kinds of pathogenic microorganisms were found by both mNGS and conventional culture. mNGS, metagenomic next-generation sequencing.

**Figure 3:**
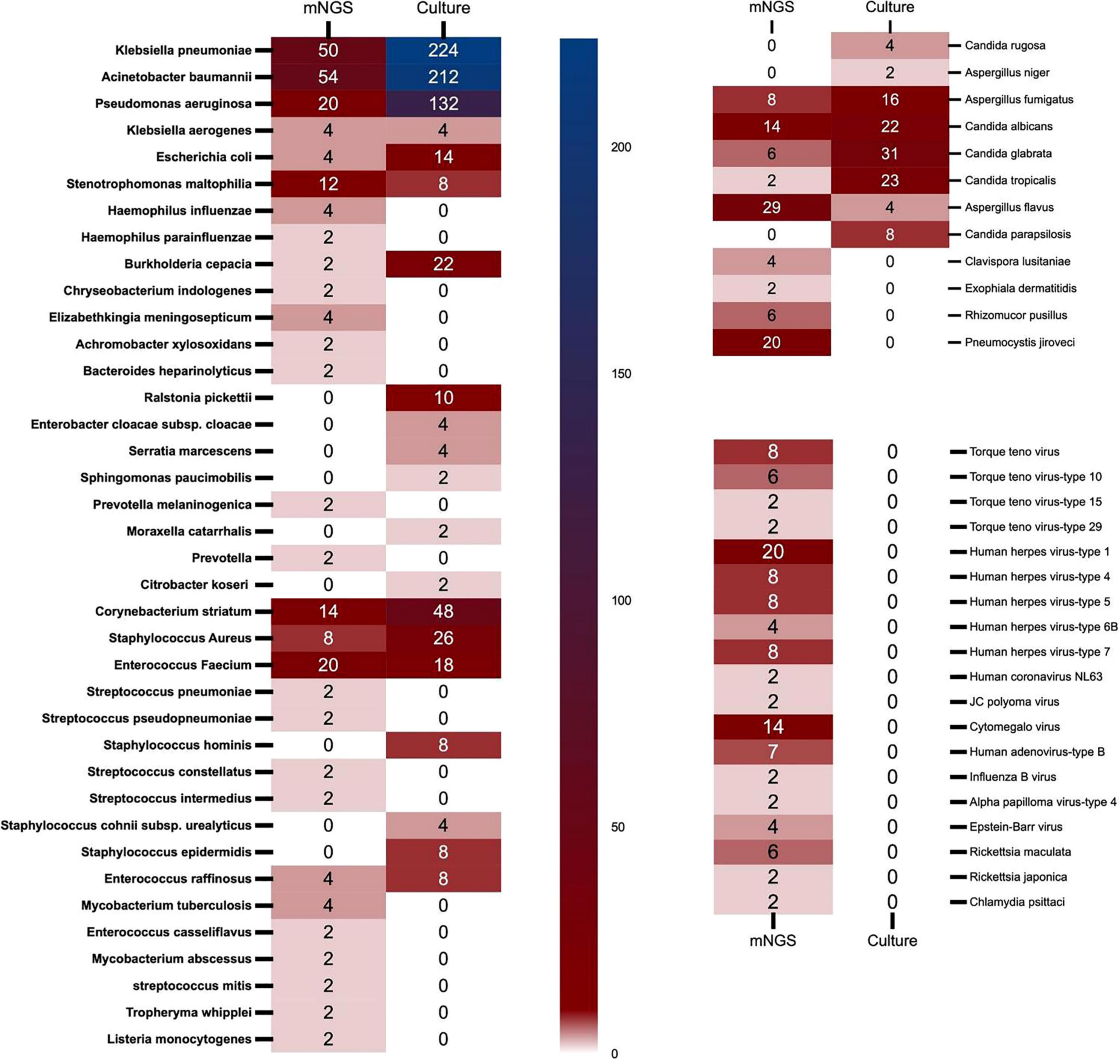
The heat map of pathogenic microorganisms details in different methods (mNGS and conventional culture). mNGS, metagenomic next-generation sequencing.

Of the 62 enrolled ECMO patients, 103 samples were submitted for mNGS detection, including 37 (35.9%) blood samples and 56 (54.4%) bronchoalveolar lavage fluid (BALF) samples. Of the above samples, 82 (79.6%) were positive. However, 1,249 samples were successively sent for traditional culture, of which 380 (30.4%) were positive. Although the number of samples submitted for examination by traditional culture was more than that for mNGS detection, the types of pathogenic microorganisms cultured were less than those detected by mNGS (28 *vs.* 58). The spectrum of pathogenic microorganisms detected by the two methods was not identical because only 17 pathogenic microorganisms can be detected by both detection methods (**Figure 2**). Traditional culture cannot cultivate viruses and special microorganisms (such as *Rickettsia* and *Chlamydia*) that can be detected by mNGS (**Figure 3**).

### Pathogenic microorganisms in different treatment periods

**Figure 4:**
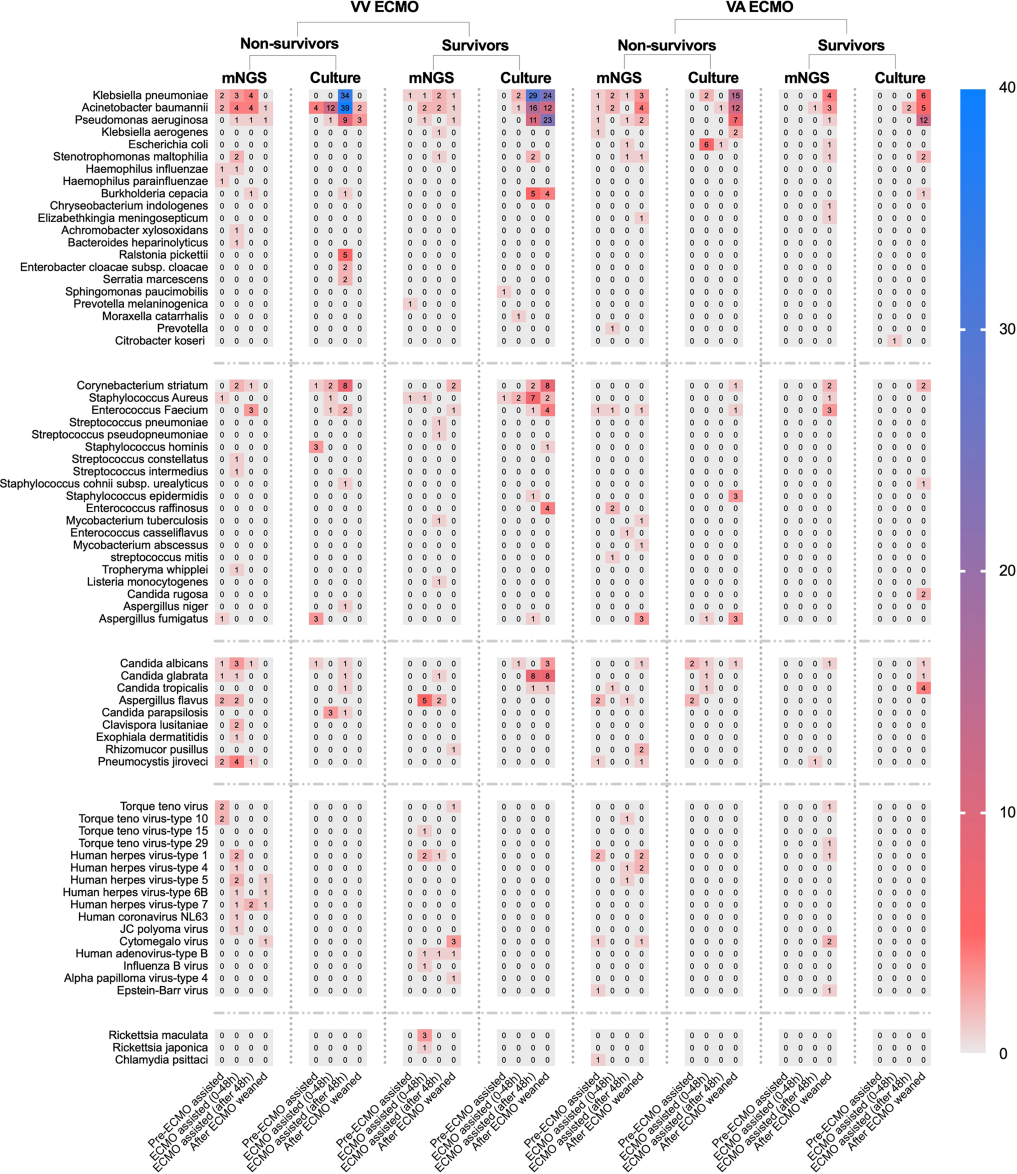
The heat map of pathogenic microorganism details in non-survivors and survivors of different ECMO assistance types (VV ECMO and VA ECMO) in different time periods (before ECMO assistance, within 48 h of ECMO assistance, after 48 h of ECMO assistance, and after weaning of ECMO). ECMO, extracorporeal membrane oxygenation; VV, veno-venous; VA, veno-arterial.

**Figure 5:**
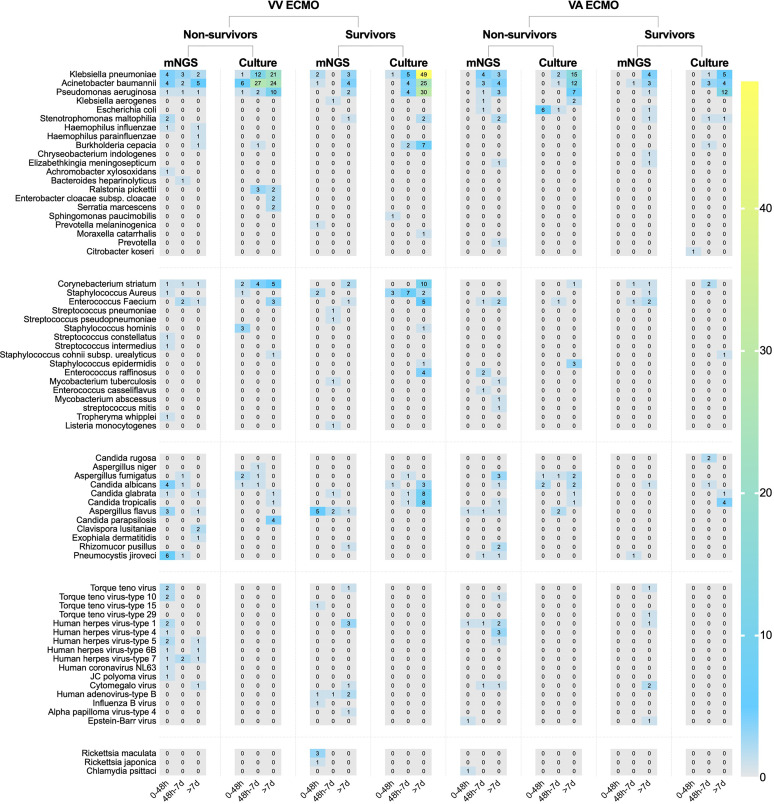
The heat map of pathogenic microorganism details in non-survivors and survivors of different ECMO assistance types (VV ECMO and VA ECMO) in different time periods (within 48 h after admission, between 48 h and 7 days of admission, and after 7 days of admission). ECMO, extracorporeal membrane oxygenation; VV, veno-venous; VA, veno-arterial.

In conventional culture, the most frequent Gram-negative bacteria, Gram-positive bacteria, and fungi were *Klebsiella pneumoniae*, *Corynebacterium striatum*, and *Candida glabrata*. Those with the highest frequency of occurrence of mNGS detection were *Acinetobacter baumannii*, *Enterococcus faecium*, and *Aspergillus flavus* (**Figure 3**). Moreover, the human herpes virus was the most prevalent virus detected by mNGS in ECMO patients (**Figure 3**). **Figure 4** shows the details of pathogenic microorganisms in non-survivors and survivors of different ECMO assistance types (VV ECMO and VA ECMO) in different time periods (before ECMO assistance, within 48 h of ECMO assistance, after 48 h of ECMO assistance, and after weaning of ECMO). It can be seen from **Figure 4** that the mNGS detection technology detected pathogenic microorganisms earlier than traditional culture in VV ECMO-assisted patients. In addition, mNGS can help diagnose special infections, including mixed infections and infections caused by special pathogens. In **Figure 5**, to avoid some patients assisted by ECMO only in the late stage of treatment, the time period has been re-divided into three different times: 48 h after admission, between 48 h and 7 days of admission, and after 7 days of admission. Combining **Figures 4**, **5**, it can be seen that patients supported by VA ECMO usually undergo detection of pathogenic microorganisms later. In addition, although the sensitivity of mNGS was high, the times of traditional culture were significantly more than those of mNGS detection.

## Discussion

In this single-center study involving non-organ transplantation critically ill patients assisted with ECMO, we once again verified that the effectiveness and comprehensiveness of mNGS detection are higher than those of traditional culture, and we found that the traditional culture and mNGS detection are inconsistent. More importantly, we found that different types of ECMO and different treatment periods have different pathogenic microorganisms and different clinical tendencies of detection methods.

ECMO is a percutaneously implantable mechanical circulatory assisting technology, which has the advantages of convenient insertion independent of location and simultaneous dual ventricular combined breathing assistance. Therefore, in recent years, it has been applied in cases of acute circulatory and/or respiratory failure where conventional life support is ineffective (Mandawat and Rao, 2017). The operations of ECMO preparation, boarding, and weaning have all been rapidly improved in practice, and the survival rate of critically ill patients has been greatly improved as a result. However, the mortality rate of ECMO patients is still very high, and the reasons are considered to be closely related to the patients’ primary disease, complications, infection, and other factors. Infections could increase the morbidity and mortality of hospitalized critically ill patients. The incidence of infections in ECMO patients reported in the literature varies widely, fluctuating between 20% and 60% (Bizzarro et al., 2011; Schmidt et al., 2012; Austin et al., 2017; Grasselli et al., 2017). All ECMO patients included in this article had definite infections during hospitalization. In clinical work, the cultivation and detection of pathogenic microorganisms are often carried out with the change of the disease, which is different from well-planned clinical trials. Therefore, mNGS detection and traditional culture in the real world may provide a more realistic reference and help guide the clinical application of various pathogen detection methods.

Our research results found that in conventional culture, the most frequent Gram-negative bacteria, Gram-positive bacteria, and fungi were *K. pneumoniae*, *C. striatum*, and *C. glabrata*. Those with the highest frequency of occurrence of mNGS detection were *A. baumannii*, *E. faecium*, and *A. flavus*. Of course, this conclusion would be interfered with to some extent because traditional culture is inexpensive and usually repeated using different samples. It can also be seen from this article that there were 103 samples of mNGS and 1,249 samples of traditional culture. The positive rate of traditional culture (30.4%) was lower than that of mNGS detection (79.6%), which was consistent with previous studies (Cheng et al., 2019). Then, we found that mNGS detected various viruses, *Rickettsia*, *Chlamydia*, *Mycobacterium*, *Tropheryma whipplei*, and *Listeria monocytogenes*. These microorganisms were clinically confirmed as pathogens, which again confirmed the necessity of mNGS detection in special pathogen infection and mixed infection. Then, after dividing the patients in detail according to the length of stay, we found that some of the results of mNGS detection were earlier than those of the traditional culture, and the infection of VV ECMO patients was significantly earlier than that of VA ECMO patients, which may be due to the infection of VV ECMO patients before the application of ECMO, and the infection rate of arterial ECMO cannulas was lower than that of venous ECMO cannulas.

The technology of mNGS has already been widely used in clinical practice. For the diagnosis of infectious diseases, mNGS is highly sensitive, rapid, and widely available (Brown et al., 2018; Carpenter et al., 2019). In addition, mNGS does not require the isolation of pathogenic bacteria and is not affected by the use of antibiotics, thus reducing the false-negative rate. Notably, mNGS assay can theoretically detect all the nucleotide sequences present within a given sample, allowing for the simultaneous detection of multiple pathogens. A recent study found that mNGS samples could provide more accurate diagnostic information for infections (Chen et al., 2021). In the case of concomitant prophylactic antibiotic treatment, early mNGS may accurately guide treatment regimens. In patients whose causative organism remains elusive, we envisage that the incidence of infections will be lowered by reducing the duration of prophylactic antibiotic application. Meanwhile, patients in whom the suspected causative organism has been detected may benefit from a more precise and individualized form of treatment. Thus, mNGS is likely to prevent the development of infections caused by antibiotic abuse. There are many advantages of mNGS detection. However, traditional training is still indispensable. In our single-center study, 39.3% of the pathogens in traditional culture were not detected by mNGS. The combination of traditional culture and mNGS detection to find pathogens and conduct drug sensitivity tests is more helpful to guide clinical treatment in the real world.

This study was a single-center retrospective study with a small sample size. Thus, a large, multi-center, prospective randomized controlled study is needed to further verify the results.

## Conclusion

During the treatment of severe patients supported by ECMO, there were different kinds of pathogenic microorganisms infected in different treatment stages and different types of ECMO. The combination of multiple applications of mNGS detection and traditional culture could improve the detection rate of pathogenic microorganisms.

## Data availability statement

The data that support the findings of this study have been deposited into EMBL database with accession number PRJEB57970.

## Ethics statement

The studies involving human participants were reviewed and approved by the Ethics Committee of the First Affiliated Hospital of Zhengzhou University. The patients/participants provided their written informed consent to participate in this study. Written informed consent was obtained from the individual(s) for the publication of any potentially identifiable images or data included in this article.

## Author contributions

XZ and L-PB: software, formal analysis, data curation, and writing—original draft. XZ, Y-CZ and B-YL: conceptualization, methodology, and writing—review and editing. Y-CZ and Z-ZY: validation and formal analysis. X-YZ: term, resources, project administration, and funding acquisition. All authors contributed to the article and approved the submitted version.
